# Supplementary material for: Schmallenberg virus non-structural proteins NSs and NSm are not essential for experimental infection of Culicoides sonorensis biting midges
Source: J Virol. 2025 May 8;99(6):e00343-25. doi: 10.1128/jvi.00343-25 (PMC12172452; doi:10.1128/jvi.00343-25)

**Supplementary Material**

**Supplementary Figure S1:** Comparison of the protein-coding regions of the S-segment (upper panel), M-segment (middle) and L-segment (lower panel) sequences of the SBV strains used in this study. The sequences of the wild-type (wt) strain SBV BH80/11-4 are used as reference. Nucleotide (orange bar alongside the name of each SBV variant) and amino acid (blue bars) substitutions are highlighted as vertical black lines. For the S‑segment, the amino acid sequences are given separately for the N-protein (light blue) and for the non-structural protein NSs (dark blue), which is encoded in an alternative overlapping reading frame. Red triangles indicate mutations within the three potential translation start codons of the NSs-encoding sequence that abrogate the expression of the NSs-protein.

**
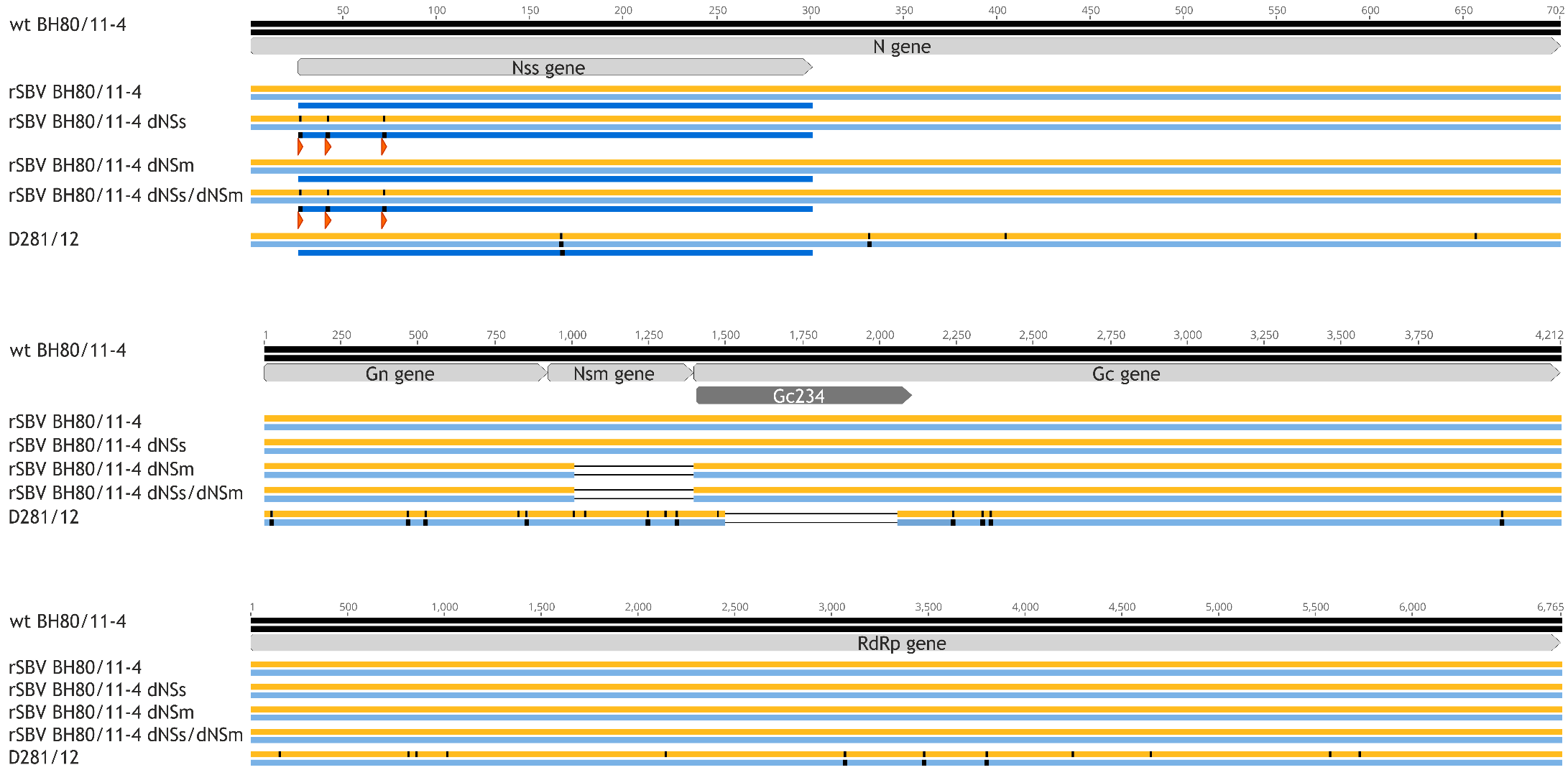
**

**Supplementary Figure S2:** Results of virus isolation in cell culture in relation to the RT-qPCR results. A randomly taken subset of 16 midges each from the negative control, the BH80/11-4 and D281/12 groups collected immediately after feeding, and all PCR-positive midges collected at day 6 from the BH80/11-4 and D281/12 groups were subjected to virus isolation using BHK cells. Red triangles correspond to midges from which virus could be re-isolated, while black triangles represent negative virus isolation results.


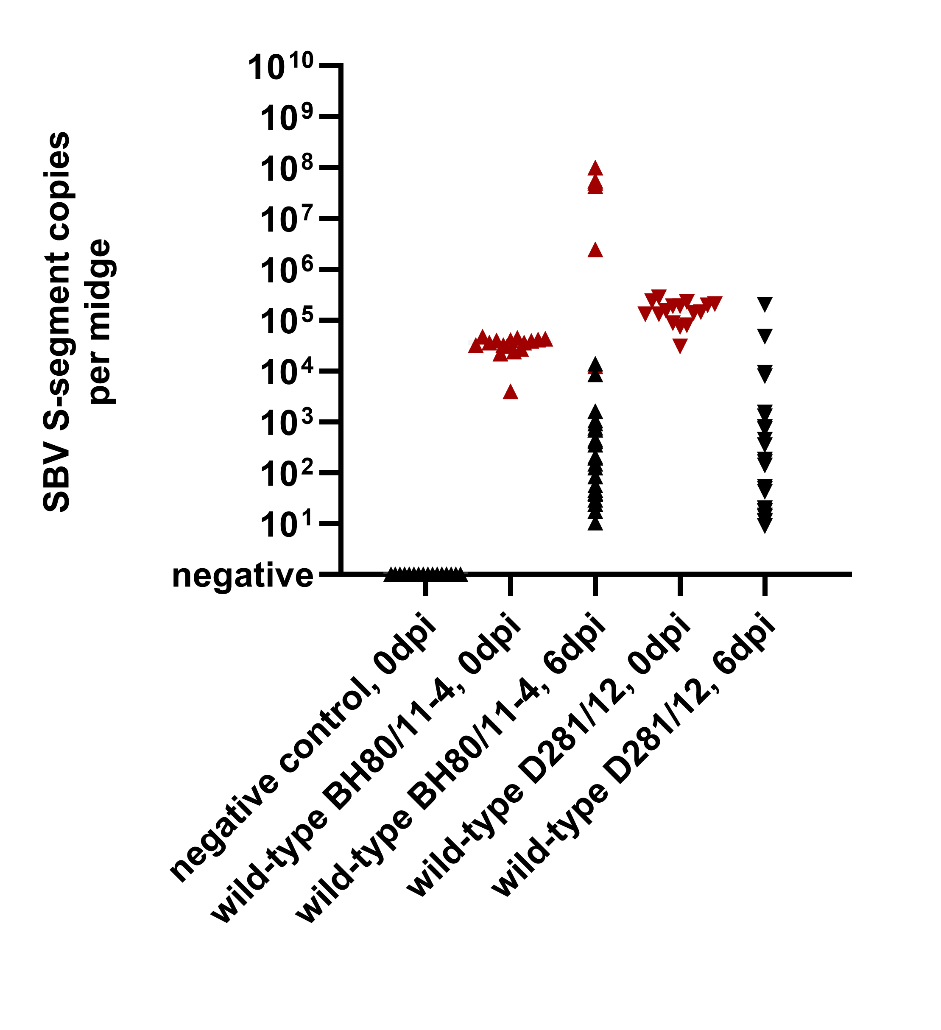

Supplement: Supplemental material — Figures S1 and S2. [file jvi.00343-25-s0001.docx]
